# Clinical outcomes and predictive factors of microsurgical varicocelectomy in men with idiopathic non-obstructive azoospermia or cryptozoospermia: a retrospective cohort study

**DOI:** 10.7717/peerj.21904

**Published:** 2026-11-10

**Authors:** Hailin Huang, Songxi Tang, Huiliang Zhou

**Affiliations:** Department of Andrology and Sexual Medicine, The First Affiliated Hospital of Fujian Medical University, Fuzhou, Fujian Province, China

**Keywords:** Non-obstructive azoospermia, Varicocele, Follicle-stimulating hormone, Male infertility, Microsurgical varicocelectomy

## Abstract

**Objective:**

To evaluate clinical outcomes and non-invasive predictors of ejaculated sperm recovery after microsurgical varicocelectomy in men with idiopathic non-obstructive azoospermia (iNOA) or cryptozoospermia.

**Methods:**

We retrospectively analyzed the clinical data of patients with iNOA or cryptozoospermia and varicocele at the First Affiliated Hospital of Fujian Medical University from January 2017 to January 2024. All patients were concurrently diagnosed with clinical varicocele via combined Doppler ultrasound and physical examination, and all underwent subinguinal microscopic varicocelectomy. A total of 30 patients were included in the study and followed-up for at least 1 year. Patients were divided into two groups according to their postoperative outcomes. The differences in various parameters between the two groups were compared. Univariate logistic regression was used to calculate the odds ratio (OR) and 95% confidence interval (95% CI) for each predictor. The logistic regression model was used to evaluate the association between sperm recovery and clinical factors. The receiver operating characteristic (ROC) curve was used to evaluate the accuracy of predictive factors.

**Results:**

Among the 30 patients who received microsurgical varicocelectomy, motile sperm was detected in postoperative ejaculates in nine patients (30.0%). Serum follicle-stimulating hormone (FSH) and luteinizing hormone (LH) levels differed significantly between the two groups (*P* < 0.05). Binary logistic regression analysis of age, mean testicular volume, FSH, LH and testosterone (T) levels showed that only FSH was associated with sperm recovery after surgery, while FSH was negatively associated with sperm recovery (OR = 0.587, 95% CI [0.345–0.998], *P* = 0.049). There was no statistically significant correlation between the other parameters. ROC analysis showed that preoperative serum FSH level could effectively predict sperm recovery after surgery (area under curve (AUC) = 0.931, 95% CI [0.805–1.000]). The sensitivity of FSH was 95.2%, the specificity was 88.9%, and the cutoff value was 10.86.

**Conclusions:**

Microscopic varicocelectomy for the treatment of iNOA or cryptozoospermia may benefit a subset of patients, with motile sperm appearing in the non-centrifuged semen postoperatively. Preoperative serum FSH level serves as an independent predictive biomarker for postoperative sperm recovery among men with iNOA. Lower baseline FSH values correlate with a greater probability of detectable motile sperm in ejaculate after microsurgical varicocelectomy.

## Introduction

Non-obstructive azoospermia (NOA) refers to testicular failure in men with severe defects in spermatogenesis, resulting in the absence of sperm in the ejaculated semen. The common causes of NOA include Y chromosome microdeletion, Klinefelter’s syndrome, adolescent cryptorchidism, mumps or orchitis after infection, gonadal toxin exposure, testicular trauma, *etc*. Some patients with NOA have no clear cause, which is called idiopathic (Fretz & Sandlow, 2002). In male related factors, about 10% of cases are diagnosed with NOA, which is the most severe type (Jarow, Espeland & Lipshultz, 1989).

Microsurgical testicular sperm extraction (M-TESE) combined with intracytoplasmic sperm injection (ICSI) can bring hope for NOA patients to obtain their own genetic offspring (Schlegel et al., 1997). So far, there is no definitive and effective treatment for NOA. In 1952, Tulloch first reported the improvement of semen parameters in a couple with NOA after varicocele repair, resulting in natural pregnancy for the partner (Tulloch, 1955). Since then, scholars have conducted extensive research on semen parameters, sperm retrieval rate (SRR), and pregnancy rate after varicocele treatment in NOA patients. Regrettably, there is still controversy over whether surgery benefits such patients. According to the American Urological Association/American Society for Reproductive Medicine (AUA/ASRM) guideline statement on men with NOA and varicocele, the couple should be advised that there is a lack of clear evidence to support varicocele repair before assisted reproductive technology (Schlegel et al., 2021). The European Association of Urology (EAU) guidelines state that in men with NOA and varicocele, varicocele repair can result in sperm in the ejaculate of 20.8% to 55% of men. They also state that varicocele repair is associated with an increased rate of surgical sperm retrieval in cases where sperm is not returned to the ejaculated semen. The EAU statement also explicitly cautions that these data come from observational studies and recommends discussing the risks and benefits of varicocele repair with men with clinically palpable varicocele and NOA (Minhas et al., 2021).

Although there are many reasons for NOA, the possibility of azoospermia due to severe testicular failure due to varicocele is present (Practice Committee of the American Society for Reproductive Medicine in collaboration with the Society for Male Reproduction and Urology, 2018). In clinical reproductive practice, varicocele and NOA often co-occur. They may have a causal relationship or just be a coincidence. However, after a detailed evaluation to rule out other causes of azoospermia, it is appropriate to discuss varicocele repair with patients as the next step (Kavoussi, Gupta & Shah, 2025). A recent global survey showed that 57.7% of surgeons would recommend varicocele repair (Rambhatla et al., 2025). Therefore, many scholars have tried to find a reliable predictor of sperm recovery for a long time.

This study aims to analyze the factors that may affect sperm recovery in iNOA or cryptozoospermia patients and evaluate the value of preoperative non-invasive factors in sperm recovery.

## Materials and Methods

### Study subjects

Clinical data were retrospectively obtained from patients enrolled between January 2017 and January 2024. Data collection approximately commenced on June 1, 2022, and ended on March 1, 2025. The clinical data of 30 patients were collected. Each patient was carefully asked for medical history, strict physical examination, and the patient’s testicular volume, body mass index (BMI), age, sex hormones, pathological types and other indicators were collected. The Prader orchidometer was used to evaluate the testicular volume. The average volume of bilateral testicles was used as the analysis index. On the day of surgery, all patients simultaneously underwent subinguinal microsurgical varicocelectomy and diagnostic testicular core biopsy. Postoperative histopathological specimens were used to quantitatively evaluate the severity of spermatogenic impairment and predict the risk of postoperative sperm recovery. All patients were followed-up for a minimum of 12 months with standardized semen analysis performed 2–3 times during follow-up. No secondary postoperative M-TESE was routinely scheduled for patients without detectable sperm at the 12-month follow-up. All surgical procedures were performed by a single surgeon with more than 10 years of experience in andrological microsurgery, who had completed standardized advanced microsurgical training for male infertility. This study was approved by the Ethics Committee of the First Affiliated Hospital of Fujian Medical University (Approval No.: [2020]375). All procedures performed in this study were in accordance with the ethical standards of the institutional research committee and with the 1964 Helsinki Declaration and its later amendments. Written informed consent was obtained from all patients prior to surgery, which included explicit consent for the use of their clinical data in this retrospective research. All patient data were fully anonymized and de-identified before analysis, with all personal identifiers removed to protect patient privacy, and the data could not be traced back to individual subjects.

#### Clinical indications and preoperative counselling

All patients were confirmed to have clinical varicocele through rigorous physical examination and Doppler ultrasonography, with comprehensive examinations performed to exclude secondary azoospermia. Patients declined upfront M-TESE combined with ICSI and opted for microsurgical varicocelectomy *via* shared physician-patient decision-making.

Standardized preoperative counselling covered postoperative sperm recovery rates cited in EAU guidelines, potential surgical complications, and alternative fertility treatments. Every patient signed written informed consent after being fully informed of all surgical risks and benefits.

#### Inclusion criteria

1. Patients were diagnosed with complete azoospermia or cryptozoospermia based on at least three sperm quality analyses performed preoperatively with an interval of more than 2 weeks between each analysis; 2. Patients had unilateral or bilateral clinical varicocele; 3. Patients were aged younger than 40 years.

#### Exclusion criteria

1. Obstructive azoospermia caused by obstruction in the epididymis, vas deferens, ejaculatory orifice, *etc.*; 2. Congenital cryptorchidism; 3. Genetic abnormalities such as peripheral blood chromosomal abnormalities and Y chromosome microdeletions; 4. Factors damaging testicular spermatogenic function, such as orchitis, epididymitis, trauma, radiotherapy, chemotherapy, *etc*.

## Research Methods

Semen examination required sexual abstinence for 2–7 days. Specimens were collected by masturbation into uniform sterile semen collection cups and processed according to the procedures and standards specified in the 6th edition of the World Health Organization Laboratory Manual for the Examination and Processing of Human Semen. Complete azoospermia was defined as the absence of sperm in semen after centrifugation at 3000 ×g for 15 min in at least three semen analyses. Cryptozoospermia represents a distinct clinical condition, not a subtype of azoospermia, and was defined as no sperm observed in uncentrifuged semen, with occasional sperm detected in the centrifuged semen pellet. Sperm recovery was defined as the presence of motile sperm in uncentrifuged semen in at least one semen examination within 1 year after surgery.

Peripheral venous blood was collected after overnight fasting between 08:00 and 11:00 for reproductive hormone measurement. Laboratory-specific reference ranges for each hormone were as follows: Prolactin (PRL) was 86–324 mIU/L; follicle-stimulating hormone (FSH): 1.5–12.4 IU/L; luteinizing hormone (LH): 1.7–8.6 IU/L; testosterone (T): 8.64–29.0 nmol/L; estradiol (E2): 41.4–159 pmol/L.

Microscopic Varicocelectomy Procedure: The patient was placed in a supine position. An oblique incision along the skin lines was made below the external ring, dissecting through the skin, subcutaneous tissue, and down to the superficial fascia. The spermatic cord was extracted from the incision, with the vas deferens and its accompanying arteries and veins preserved and separated by a rubber sheet. Vascular clamps were applied on both sides of the rubber sheet to fix the cord, exposing the spermatic cord outside the incision. The external spermatic fascia, cremaster muscle, and internal spermatic fascia were incised longitudinally. Under the microscope, 5-0 silk sutures were used to ligate the veins running within the internal spermatic fascia, cremaster muscle, and external spermatic fascia. Arteries and lymphatic vessels within the internal spermatic fascia, cremaster muscle, and external spermatic fascia were preserved under microscopic visualization. Hemostasis was carefully achieved, and the internal and external spermatic fasciae were sutured. The spermatic cord was returned to the incision, followed by layered closure of the superficial fascia, subcutaneous tissue, and skin.

### Statistical methods

Data were processed using SPSS 22.0 statistical software. The normality of continuous variables was verified by the Shapiro–Wilk test, combined with histograms and Q–Q plots for visual judgment. Measurement data conforming to normal distribution were expressed as mean ± standard deviation (Mean ± SD), and *t*-test was used for intergroup comparison. Continuous variables showing skewed distribution were expressed as median and interquartile range (IQR), and rank sum test was adopted. Enumeration data were presented as rate (%), and Fisher’s test was used for intergroup comparison. Spearman correlation test was applied to examine the correlation among testicular volume, age, FSH, LH and T. Logistic regression model was used to evaluate the association between sperm recovery and clinical factors. The OR value and 95% confidence interval of each predictor were calculated by univariate logistic regression. We performed multivariable binary logistic regression using a backward stepwise selection approach. All clinically relevant candidate variables were initially entered into the model, followed by sequential elimination of predictors that did not reach statistical significance (*P* > 0.05). Finally, the receiver operating characteristic curve (ROC) was used to evaluate the accuracy of index test. *P* < 0.05 was considered statistically significant.

## Results

### Clinical outcomes and basic characteristics of all participants

A total of 30 patients underwent microsurgical varicocelectomy, including 25 cases of complete azoospermia and five cases of cryptozoospermia. Postoperative motile sperm was detected in ejaculate of nine patients, corresponding to an overall sperm recovery rate of 30.0% (9/30), while the remaining 21 patients exhibited no sperm after surgery (70.0%, 21/30). Stratified by preoperative azoospermia subtype, the sperm recovery rate was 24.0% (6/25) in complete azoospermia patients and 60.0% (3/5) in cryptozoospermia patients. The mean age of the whole cohort was 30.23 ± 3.18 years. Twelve patients presented with bilateral varicocele, and 18 with isolated left varicocele. Stratified by intraoperative testicular histopathology, pathological patterns were classified as hypospermatogenesis (HS), maturation arrest (MA), and Sertoli cell-only syndrome (SCOS), including four patients with HS (13.3%), 14 with MA (46.7%), and 12 with SCOS (40.0%) (Table 1).

**Table 1:** Baseline characteristics of the whole study cohort. Data are presented as mean ± standard deviation or median (interquartile range); categorical variables are shown as n (%). Total cohort *N* = 30.

**Parameter**	**Statistical Description**
Age (years)	30.23 ± 3.18
BMI (kg/m^2^)	23.11 ± 3.41
Duration of infertility (years)	4.21 ± 2.35
Testicular volume (mL)	9.62 ± 3.53
FSH (IU/L)	15.16 ± 8.70
LH (IU/L)	7.44 ± 3.82
T (nmol/L)	16.16 ± 6.63
PRL (mIU/L)	299.35 (221.26–355.50)
E2 (pmol/L)	93.14 (71.05–143.40)
Preoperative azoospermia subtype, n (%)	
Complete azoospermia	25 (83.3)
Cryptozoospermia	5 (16.7)
Varicocele laterality, n (%)	
Unilateral (left)	18 (60.0)
Bilateral	12 (40.0)
Preoperative testicular histopathology, n (%)	
Hypospermatogenesis (HS)	4 (13.3)
Maturation arrest (MA)	14 (46.7)
Sertoli cell-only syndrome (SCOS)	12 (40.0)
Smoking history, n (%)	10 (33.3)
Alcohol consumption, n (%)	16 (53.3)

**Notes.**

Abbreviations FSH follicle-stimulating hormone LH luteinizing hormone T testosterone PRL prolactin E2 estradiol HS hypospermatogenesis MA maturation arrest SCOS Sertoli cell-only syndrome

### Comparison of parameters between sperm recovery positive and negative groups

We found significant differences in serum FSH and LH levels between the two groups (*P* < 0.05). In addition, there was no significant difference in age, testicular volume, BMI, T, E2 and PRL between the two groups (*P* > 0.05) (Table 2).

**Table 2:** Comparison of postoperative sperm presence between groups. Data are presented as mean ± standard deviation or median (interquartile range); categorical variables are expressed as n (%).

**Characteristics**	**Positive group (sperm retrieved, *n* = 9)**	**Negative group (no sperm retrieved, *n* = 21)**	***P* value**
Age (years)	31.11 ± 3.02	29.86 ± 3.25	0.331
Testicular volume (mL)	10.83 ± 4.24	9.09 ± 3.14	0.222
BMI (kg/m^2^ )	23.10 ± 3.62	23.12 ± 3.40	0.989
FSH (IU/L)	6.70 ± 5.30	18.78 ± 7.25	0.001
LH (IU/L)	4.31 ± 2.01	8.79 ± 3.64	0.002
PRL (mIU/L)	356.80 (194.72, 627.05)	279.44 (197.02, 345.10)	0.248
E2 (pmol/L)	107.60 (74.36, 152.45)	92.60 (53.69, 144.85)	0.455
T (nmol/L)	16.37 ± 6.82	16.07 ± 6.71	0.913
Testicular volume stratification, n (%)			0.704
>8 mL	6 (66.7)	12 (57.1)	—
≤8 mL	3 (33.3)	9 (42.9)	—

**Notes.**

Abbreviations FSH follicle-stimulating hormone LH luteinizing hormone T testosterone PRL prolactin E2 estradiol

We stratified all patients according to testicular histopathological findings and calculated the postoperative sperm recovery rate for each subtype (Table 3). The sperm recovery rate was highest in patients with HS (75.0%), followed by MA (35.7%), while only 8.3% of patients with SCOS achieved detectable sperm after surgery. The overall distribution of pathological subtypes differed significantly between sperm recovery-positive and negative groups (*χ*^2^ = 6.757, *df* = 2, *P* = 0.0341). Patients with HS or MA predominated in the positive group, whereas SCOS accounted for the majority of the negative cohort (Table 3).

**Table 3:** Testicular histopathological subtypes and postoperative sperm recovery outcomes. Data are presented as number of patients (n).

**Histopathological subtype**	**All patients (*n* = 30)**	**Sperm retrieval positive (*n* = 9)**	**Sperm retrieval negative (*n* = 21)**	**Sperm recovery rate (%)**
HS	4	3	1	75.0
MA	14	5	9	35.7
SCOS	12	1	11	8.3

**Notes.**

Statistical analysis: Between-group differences in pathological subtype distribution were analyzed using the Pearson *χ*^2^ test. *χ*^2^ = 6.757, *df* = 2, *P* = 0.034.

Abbreviations HS hypospermatogenesis MA maturation arrest SCOS Sertoli cell-only syndrome

### Univariate regression analysis of parameters between the two groups

Univariate logistic regression analysis showed that FSH and LH levels were significantly associated with positive sperm recovery, both of which were negatively correlated with sperm recovery. The OR values were 0.69 (95% CI [0.52–0.91], *P* = 0.009) and 0.55 (95% CI [0.35–0.87], *P* = 0.01), respectively (Table 4).

**Table 4:** Univariate logistic regression analysis of clinical predictors for postoperative sperm recovery.

**Characteristic**	**OR (95% CI)**	***P* value**
Age (years)	1.14 (0.88–1.48)	0.322
Testicular volume (mL)	1.16 (0.92–1.47)	0.220
FSH (IU/L)	0.69 (0.52–0.91)	0.009
T (nmol/L)	1.01 (0.89–1.13)	0.909
LH (IU/L)	0.55 (0.35–0.87)	0.010
BMI (kg/m^2^ )	0.99 (0.79–1.26)	0.989
E2 (pmol/L)	1.00 (0.99–1.01)	0.874
PRL (mIU/L)	1.00 (0.99–1.01)	0.137

**Notes.**

Abbreviations FSH follicle-stimulating hormone LH luteinizing hormone T testosterone E2 estradiol PRL prolactin OR odds ratio 95% CI 95% confidence interval

### Analysis of preoperative independent predictors for postoperative sperm recovery

The logistic regression model was used to predict the factors of sperm recovery, including age, testicular volume, FSH, LH, and T. Ultimately, only FSH level was found to be significantly associated with positive sperm recovery, showing a negative correlation (OR = 0.587, 95% CI [0.345–0.998], *P* = 0.049). No statistical significance was observed for the remaining parameters (Table 5).

**Table 5:** Multivariate binary logistic regression analysis of predictors for postoperative sperm retrieval in patients with iNOA. β, regression coefficient; SE, standard error; Wald *χ*^2^, Wald chi-square statistic; OR, odds ratio; 95% CI, 95% confidence interval.

**Variables**	**β coefficient**	**SE**	**Wald *χ*^2^**	**OR**	**95% CI**	***P* value**
Age (years)	0.412	0.318	1.678	1.510	0.809–2.815	0.195
Testicular volume (mL)	0.115	0.253	0.207	0.891	0.543–1.464	0.649
FSH (IU/L)	0.532	0.271	3.869	0.587	0.345–0.998	0.049
T (nmol/L)	0.067	0.120	0.314	0.935	0.739–1.183	0.575
LH (IU/L)	0.088	0.303	3.863	1.092	0.602–1.979	0.773
Constant	5.717	10.201	0.314	0.003	–	0.575

**Notes.**

Abbreviations NOA non-obstructive azoospermia FSH follicle-stimulating hormone LH luteinizing hormone T testosterone

### The accuracy of FSH in predicting postoperative semen recovery was determined by ROC curve

ROC analysis showed that serum FSH level could effectively predict sperm recovery in postoperative semen (AUC = 0.931, 95% CI [0.805–1.000]). The sensitivity, specificity and cutoff value of FSH were 95.2%, 88.9% and 10.86, respectively (Fig. 1).

**Figure 1:**
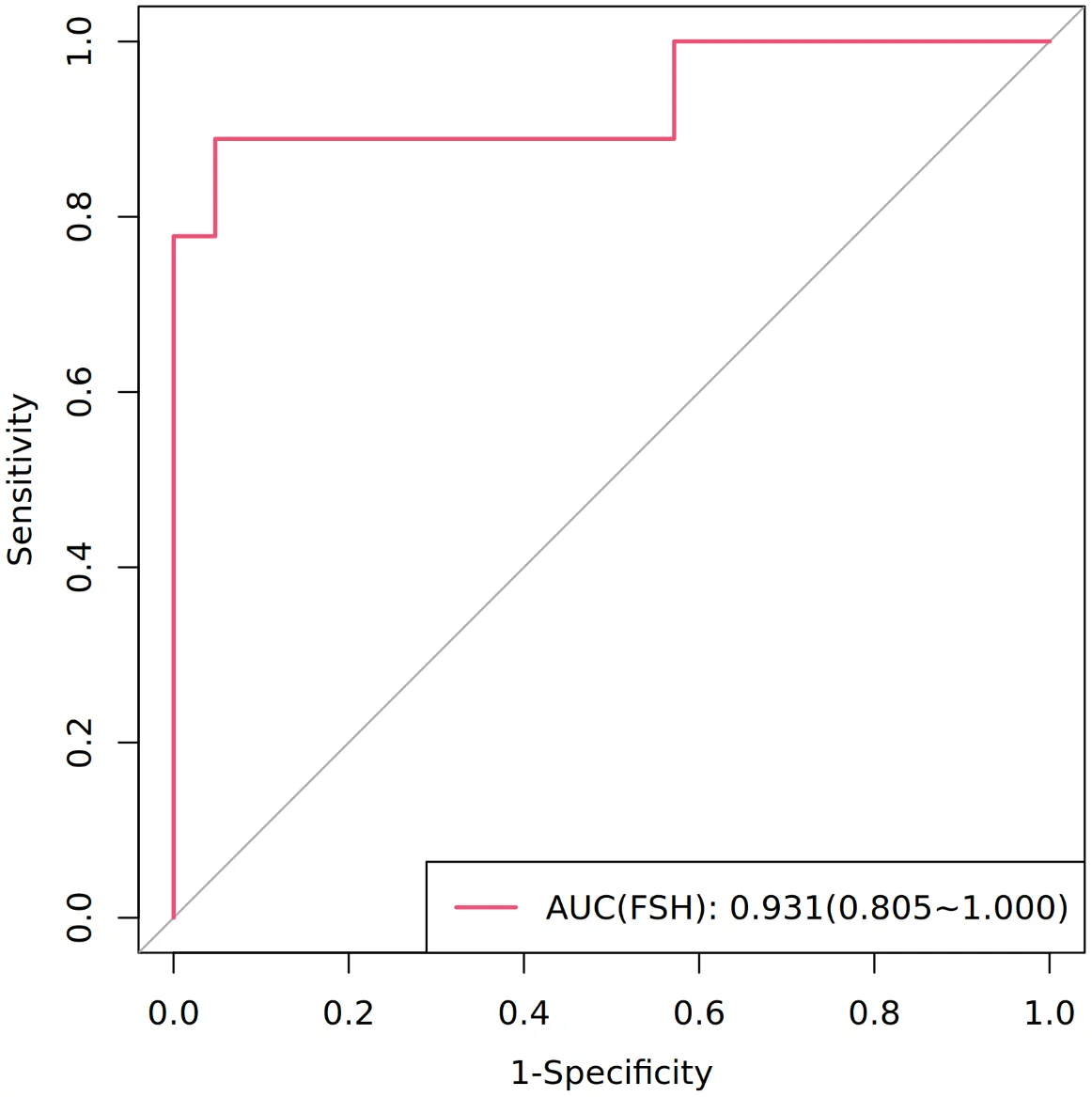
Receiver operating characteristic (ROC) curve was used to determine the accuracy of FSH in predicting postoperative semen recovery.

### Data availability

All data generated and analyzed during this study are included in the manuscript. No external or unpublished data were used. All supporting values, statistical results, baseline patient information, and predictive model data are fully presented within the tables, figures, and text of this article. There are no additional restricted data.

## Discussion

With the advent of the ICSI era, patients only need a minimal amount of sperm to have the chance to obtain genetically related offspring (Schlegel et al., 1997). In recent years, there have been increasing studies on varicocele and NOA. Cumulative data show that varicocele repair surgery can restore sperm in the semen, improve SRR and live birth rates, and even lead to spontaneous pregnancy (Schlegel & Kaufmann, 2004; Pasqualotto et al., 2006; Youssef et al., 2009; Weedin, Khera & Lipshultz, 2010; Sajadi et al., 2019; Esteves et al., 2016). In 2003, Schlegel and Kaufmann et al. evaluated 31 NOA men who had undergone varicocele repair and found that 22% (7/31) of the patients had sperm on at least one post-surgical semen examination. However, the study showed that only a few patients had enough sperm for ICSI, approximately 9.6% (Schlegel & Kaufmann, 2004). Pasqualotto et al. (2006) and Youssef et al. (2009) reported 33.3% and 36.4% of motile sperm recovered in semen after varicocele repair, respectively, suggesting that varicocele repair may be an alternative to more expensive assisted reproductive techniques. In different series of analyses of postoperative semen parameters, the reported rates of sperm recovery after varicocele repair range from 21% to 56% (Mehta & Goldstein, 2012). A recent meta-analysis showed a mean positive rate of sperm recovery in semen of 27.3%, a postoperative SRR of 48.9% in the M-TESE group and 32.1% in the untreated group, and a mean postoperative natural pregnancy rate of 5.24% (Jensen & Ko, 2021). Our findings are generally consistent with previous studies, where approximately one-third of patients benefited from surgical treatment, with sperm appearing in uncentrifuged semen in at least one postoperative semen analysis. Patients with cryptozoospermia demonstrated superior postoperative sperm recovery outcomes compared to those with complete azoospermia, a finding likely explained by the presence of residual intratesticular spermatogenic foci in cryptozoospermic men.

The adverse effects of varicocele on male fertility have been extensively studied, with complex pathogenetic mechanisms. Reportedly, the incidence of azoospermia and severe oligozoospermia in men with varicocele ranges from 4.3% to 13.3% (Czaplicki, Bablok & Janczewski, 1979). The pathophysiological mechanisms by which varicocele induces male infertility primarily include scrotal hyperthermia, hypoxia, reflux of renal and adrenal metabolites, sex hormone imbalance, and antisperm antibody formation. Varicocele causes damage to testicular Sertoli cells, Leydig cells, and seminiferous tubules, leading to reduced testosterone secretion, impaired spermatogenesis, and testicular atrophy. These factors contribute to testicular spermatogenic dysfunction, occasionally presenting as azoospermia (Jensen et al., 2017; Cozzolino & Lipshultz, 2001; Pastuszak & Wang, 2015).

In clinical practice, microsurgical varicocelectomy is routinely recommended for most men diagnosed with NOA concomitant with clinical varicocele. Nevertheless, this clinical phenotype is relatively rare, and large real-world cohort data remain scarce in the current literature, which limits robust preoperative counseling for affected couples. The present study fills this research gap by reporting a validated non-invasive FSH cut-off value and stratified prognostic data based on testicular histopathology, thereby offering evidence-based references for clinical decision-making in this challenging infertile population.

Many clinical, biochemical, and histopathological indicators have been extensively studied with conflicting results. In previous studies, testicular histopathological type appeared to be the strongest predictor (Esteves et al., 2016; Majzoub et al., 2021; Abdel-Meguid, 2012; Aboutaleb et al., 2014; Elzanaty, 2014). In NOA patients, HS or MA types have a greater chance of postoperative sperm recovery (Esteves et al., 2016; Elzanaty, 2014). Patients with HS are more likely to achieve postoperative sperm recovery than those with MA, while both groups demonstrate superior prognostic potential compared to SCOS (Esteves et al., 2016; Aboutaleb et al., 2014). The results of our study also showed that there were significant differences between the different types of pathological findings. However, testicular histopathological types have certain limitations in predicting sperm recovery after surgery. First, testicular biopsy is invasive and the distribution of testicular tissue is heterogeneous. The results of a single testicular biopsy have certain limitations in estimating the presence of spermatogenic foci in the overall testis (Esteves, 2015; Saleh, Agarwal & Farouk, 2011). Second, inter-pathologist variability exists in the interpretation of testicular histology, with up to 27% of testicular biopsy results demonstrating inconsistency (Cooperberg et al., 2005).

The value of FSH levels in predicting sperm recovery after varicocele repair in men with NOA has been controversial. In most past studies, preoperative FSH levels had limited value in predicting postoperative sperm recovery. A study by Youssef et al. (2009) enrolled 83 NOA patients, including 54 with complete azoospermia and 29 with cryptozoospermia, who underwent high varicocelectomy with lymphatic sparing. Postoperatively, no significant difference in FSH levels was observed between the two groups. Aboutaleb et al. (2014) revealed no significant differences between groups in parameters such as duration of infertility, semen volume, testicular volume, and levels of FSH, LH, and T at 3 and 6 months post-varicocele repair. Additionally, a meta-analysis demonstrated that when patients were stratified by FSH levels (above or below 10 mIU/mL), no significant intergroup differences were observed in terms of improved semen parameters or natural pregnancy rates (Weedin, Khera & Lipshultz, 2010).

Conversely, some studies have reported opposing findings. D’Andrea et al. demonstrated that FSH levels following varicocele embolization serve as a valuable predictor of sperm recovery. An FSH cutoff value of <10.06 IU/L was identified, with 82.0% case applicability, 81.8% specificity, and 83.3% sensitivity (D’Andrea et al., 2015). Shiraishi et al. found that testicular histopathological types were associated with sperm recovery, whereas age, testicular volume, varicocele grade, and FSH levels showed no significant correlation with postoperative sperm recovery. However, the study noted that FSH levels were lower in the sperm recovery group, typically <10 IU/L (Shiraishi, Oka & Matsuyama, 2017).

Our study demonstrated that preoperative serum FSH acts as an independent preoperative predictor of ejaculated sperm recovery after surgery. There was a negative correlation between FSH concentration and the probability of sperm recovery: lower baseline FSH levels corresponded to a higher likelihood of postoperative sperm reappearance. Notably, although preoperative FSH exhibited excellent discriminatory performance with an AUC of 0.931, this diagnostic accuracy should be interpreted with caution given our small sample size. Prospective, large-scale multicenter trials are required to validate the 10.86 IU/L cut-off value before its widespread clinical implementation.

Consistently, our testicular histopathological findings demonstrated that patients with residual spermatogenic foci (HS or MA) had a substantially higher probability of postoperative sperm reappearance after varicocelectomy compared with those diagnosed with SCOS, a condition characterized by complete absence of germ cells. This observation confirms that preoperative testicular histopathology serves as a critical prognostic marker for surgical outcomes in men with NOA concomitant with varicocele. However, no statistically significant difference in testicular volume was detected between patients with and without postoperative sperm recovery. We then conducted exploratory subgroup analyses dichotomized by an eight mL testicular volume cut-off. This threshold was simplified from the optimal ROC-generated cutoff of 8.17 mL established by Fu, Cui & Tang (2024) a value validated to identify severe testicular hypotrophy in patients with NOA. Nevertheless, stratification by this volume threshold still failed to yield any statistically significant intergroup differences. Accordingly, the non-invasive preoperative serum FSH level carries greater clinical significance as a predictive biomarker for postoperative sperm recovery. Both baseline FSH concentrations and testicular histopathological subtypes should therefore be fully incorporated into preoperative clinical decision-making.

Several limitations of the present study should be acknowledged. First, this was a single-center retrospective observational study recruiting all participants from one tertiary andrology center; thus, selection bias could not be fully excluded, and the follow-up protocol lacked uniform standardization. The relatively small sample size may impair the reliability of the backward stepwise multivariate logistic regression model and introduce potential overfitting bias. Consequently, the multivariate predictive results require further verification in large-scale independent external cohorts. Second, postoperative hormonal adjuvant treatments were prescribed empirically, consisting of selective estrogen receptor modulators, aromatase inhibitors and gonadotropins. Of the 30 enrolled patients, 21 received no hormonal therapy, two were administered anastrozole alone, two received tamoxifen monotherapy, four were treated with combined tamoxifen and human menopausal gonadotropin, and one took clomiphene citrate. Given the limited overall sample, uneven subgroup distribution, variable treatment courses and heterogeneous medication protocols, statistical analyses to compare spermatogenic recovery across different treatment subgroups were not feasible. Combined standardized surgical and hormonal therapy may bring greater reproductive benefits and represents a valuable avenue for subsequent investigations. Finally, while individuals with abnormal peripheral blood karyotypes and Y-chromosome microdeletions were excluded from the cohort, genetic screening for other well-established genes implicated in testicular spermatogenesis was not performed. As such, we cannot eliminate the possibility that pathogenic genetic variants underlie azoospermia in a subset of our patients.The role of preoperative serum FSH level in varicocele repair in NOA men needs to be further confirmed by well-designed prospective randomized controlled trials.

## Conclusion

In this study, we found that testicular volume, LH, T, and age were not predictors of sperm recovery after varicocele repair in iNOA or cryptozoospermia patients. Conversely, preoperative serum FSH levels exhibited predictive value: the lower the FSH level, the greater the likelihood of sperm recovery.

## Supplemental Information

10.7717/peerj.21904/supp-1 Supplemental Information 1 Original data

## Additional Information and Declarations

### Data Availability

The following information was supplied regarding data availability: The data is available in the Supplemental File.
